# Beta Glucan: Supplement or Drug? From Laboratory to Clinical Trials

**DOI:** 10.3390/molecules24071251

**Published:** 2019-03-30

**Authors:** Vaclav Vetvicka, Luca Vannucci, Petr Sima, Josef Richter

**Affiliations:** 1 Department of Pathology and Laboratory Medicine, University of Louisville, Louisville, KY 40292, USA; 2 Department of Immunology, Institute of Microbiology, 142 20 Prague, Czech Republic; vannucci@biomed.cas.cz (L.V.); sima@biomed.cas.cz (P.S.); 3 Zdravotní ústav se sídlem v Ústí nad Labem, 400 01 Ústí nad Labem, Czech Republic; josef.richter@zuusti.cz

**Keywords:** glucan, drug, immunity, food, supplement, infection, mushrooms

## Abstract

Glucans are part of a group of biologically active natural molecules and are steadily gaining strong attention not only as an important food supplement, but also as an immunostimulant and potential drug. This paper represents an up-to-date review of glucans (β-1,3-glucans) and their role in various immune reactions and the treatment of cancer. With more than 80 clinical trials evaluating their biological effects, the question is not if glucans will move from food supplement to widely accepted drug, but how soon.

## 1. Why Dietary Supplements

Despite a long history of research, the question of why to use dietary supplements remains. The answer is that nutrition and infection, as well as our health in general, are intimately related [1]. The oldest research in nutritional immunity focused mainly on determining the optimal composition of nutrients and used this information particularly in the prevention of infectious diseases and to support the medical treatment of ongoing diseases. Gradually, experts began to understand that every infectious disease is accompanied by a reduction of food intake and the eventual changes in food composition, both of which result in substantial changes or even inhibition of numerous immune functions.

In cellular immunity, there are attenuated and delayed hypersensitivity reactions, a decreased number of T lymphocytes, and phagocytic dysfunction. Effects on humoral immunity were seen as a decrease in the secretion of IgA and antibodies with significant effects on their affinity, and also as a lower activity of various complement components [2]. The relationship between immunity and nutrition is considered completely interconnected [3]. Immunity is often seriously compromised during malnutrition and starvation. On the other hand, an abundance of food can also have adverse effects on immunity and the subsequent increase in the susceptibility to infectious and other noncommunicable diseases [4]. In addition, there are many infectious diseases that are accompanied by malabsorption of nutrients, which is not surprising, as malnutrition is considered to be the main cause of immunodeficiencies. Diseases connected with bad nutrition include bacterial, viral and protozoan enteritis, helminthiases, and febrile diseases, which in all cases result in malabsorption of protein, vitamins, and trace elements and subsequent catabolic loss. It is important to note that the first defensive body response to infection results from formation of the anorexia cytokines. Further, cytokines (IL-1, IL-6, IL-8, and TNF-α) are molecules which can decrease food intake [5].

The conclusions of the studies published from 1966 until 2006 related to nutritional support of anti-infectious immunity via food supplementation by various vitamins and multivitamins, trace elements, and probiotic yogurts strongly support the assumption that therapeutic effects are not unambiguous and nutritional supplements play no supportive role in the treatment of certain ongoing infections [6]. Subsequently, a new approach appeared—the possibility of immunomodulation by food. Clearly, multivitamins alone were not enough.

## 2. Glucans in the Immunity

It is known that people in prehistoric time were aware that some mushrooms may have healing properties. An ancient text found in India, written approximately 5000 years ago, discusses the medicinal effects of mushrooms, and a Japanese legend described monkeys without cancer or any other disease after feasting on the mushroom *Lentinula edodes*. Much later, Japanese interest in chemical components was based on this legend. African shamans and Native Americans used similar knowledge [7].

An immunomodulator is defined as the substance capable of interacting with the immune system resulting in up- or down-regulating specific parts of the immune response. Generally, immunomodulators represents a diverse array of synthetic, natural, and recombinant molecules, some of which were already approved for human medicine. Among these molecules, we can find almost every natural molecule imaginable including curcumin, thyme, bay leaf, resveratrol, ellagic acid, ginseng, *Echinacea*, *Aloe vera*, *Astragalus*, Goldenseal, flavonoids and essential oils, to name just a few. Many of these molecules, however, are referred by only limited scientific studies. In the last two decades, direct comparison studies of individual immunomodulators have been extremely limited, but glucans consistently showed the highest biological effects [8]. With over 20,000 published studies, glucan has the best position among other immunomodulators.

β-1,3-Glucans (hereafter referred to as glucan) are natural molecules able to significantly improve our health. They represent highly conserved structures often named pathogen-associated molecular patterns (PAMPs) (for review, see Zipfel and Robatzek [9]). A proper history of polysaccharides referred to as immunomodulators can be traced to the 1940s, when Shear and Turner [10] described a polysaccharide substance isolated from the seaweed *Serratia marcescens*, that caused tumor necrosis. The first attempt to solve the structure of this so-called Shear’s polysaccharide was done by Rathgeb and Sylvén [11]. On the basis of methylation analysis and periodate oxidation, they suggested that the polysaccharide was composed of glucose residues. It is important to note, however, that immunomodulatory preparations from bacteria, either extracts from intact cells or from isolated products, can be dangerous, which explains the focus on polysaccharides isolated from safer organisms such as yeasts, mushrooms, or even bananas [12]. During the evolution of the immune reactions, multicellular organisms developed the ability to recognize those molecules as non-self and subsequently react via various immune mechanisms. Therefore, the ability to recognize glucans is encoded by all species, from invertebrates to humans [13,14].

Further, the Di Luzio’s group evaluated the functional activity of the monocyte-macrophage system tested by clearance of colloidal carbon, and by the induced reticuloendothelial hyperplasia after the intravenous injection of various constituents of zymosan [15]. Zymosan, which is prepared from yeast, is a reagent that has been widely used for many years in inflammation and immunology. Since the main component of the yeast cell wall is glucan, it was thought that the activity of zymosan might be due to the receptor of glucan, or to the activation of the complement system by glucan. After zymocel, which is pure glucan derived from zymosan, was isolated, researchers were even more convinced that glucan plays a role in the activity of the crude zymosan. These studies found that the mannan component of the yeast cell was inactive and the activity was still present in the zymosan molecule after the removal of lipids. Additionally, the glucan application resulted in a significant reticuloendothelial activation and caused significant hyperplasia.

The relationship between structure and function remains unclear. In connection with biological activities of β-(1,3)-glucans, Bohn and BeMiller have published a survey devoted to knowledge accumulated about structure - functional activity relationships [16]. They emphasized the importance of the β-(1,3)-glucan backbone but also many noticeable contradictory data which were previously published on the influence of molecular weight, water solubility, degree of 6-*O*-substitution by glucopyranosyl units, global chain conformation and intermolecular associations on antitumor activity and on mechanisms involved by these glucans. However, no direct conclusion has been reached. Authors seeking more details on this subject should read Novak and Vetvicka [17].

For a long time, there was a controversy regarding the way of application, with only intraperitoneal or intravenous application being considered adequate. Subsequent experiments showed that orally-given glucan is active, similar to an injected dose [18]. Some detailed studies have revealed that after administering oral β-glucan (either soluble glucan or particulate glucan) for three days, labeled β-glucan within splenic macrophages appeared to be the same size as the starting material. With time, glucan aggregates were found to be concentrated at the edges of the cytoplasm near the cell membrane. Subsequent studies using a cultured macrophage cell line examined the fate of labeled glucan added to the cells. These experiments showed that phagocytosed glucan was slowly degraded within cells and that soluble biologically highly-active fragments of glucan were released into the surrounding cells. Complete macrophage degradation of glucan required approximately thirteen days. Usually, glucan particles remained intact for four days and appeared to fragment into smaller parts and soluble material during ensuing days [19]. Similar results were later independently reached by Chan’s group [20]. Less clear is the situation regarding the dose. In human studies, the tested (and suggested) daily dose remains in the range of 100–500 mg for stimulation of the immune system, whereas for a decrease in cholesterol levels a daily dose of 3 g is recommended. For comparison, the LD_50_ dose for lentinan was found to be over 2500 mg [21].

Moreover, original glucan studies focused on the effects on the murine immune system. In fact, subsequent research established that glucan has strong immunostimulating effects in a wide variety of species including earthworms, bees, shrimp, fish, chicken, rats, rabbits, guinea pigs, sheep, goat, pigs, cows, monkeys, and humans [22,23,24]. It has been concluded that glucans represent a rather special immunostimulant that is active in every species, and therefore it is one of the few immunostimulants active across the evolutionary spectrum. Some experiments even demonstrated that glucan helped in the protection of some plants [25,26].

Glucans are considered to be strong activators of cellular immunity, with macrophages being the most important biological targets. Based on the first wave of studies, glucan has been established in protection against infection. The protective effects of glucan application were shown in experimental models of infection with *Leishmania major*, *L. donovani*, *Candida albicans*, *Toxoplasma gondii*, *Streptococcus suis*, *Plasmodium berghei*, *Staphylococcus aureus*, *Escherichia coli*, *Mesocestoides corti*, *Trypanosoma cruzi*, *Eimeria vermiformis*, and *Bacillus anthracis* [27,28,29,30,31,32,33,34,35,36,37,38].

The attention of scientists focused on the fight against cancer. Since the original study published more than 35 years ago, the antitumor activity of glucan has been published [39]. In fact, numerous animal and human studies have shown remarkable activity against a wide variety of tumors [19,40,41,42,43]. Further, more recent studies have repeatedly shown that glucan has a strong synergy with the antibodies which naturally occur in cancer [19,44,45].

**Figure 1:**
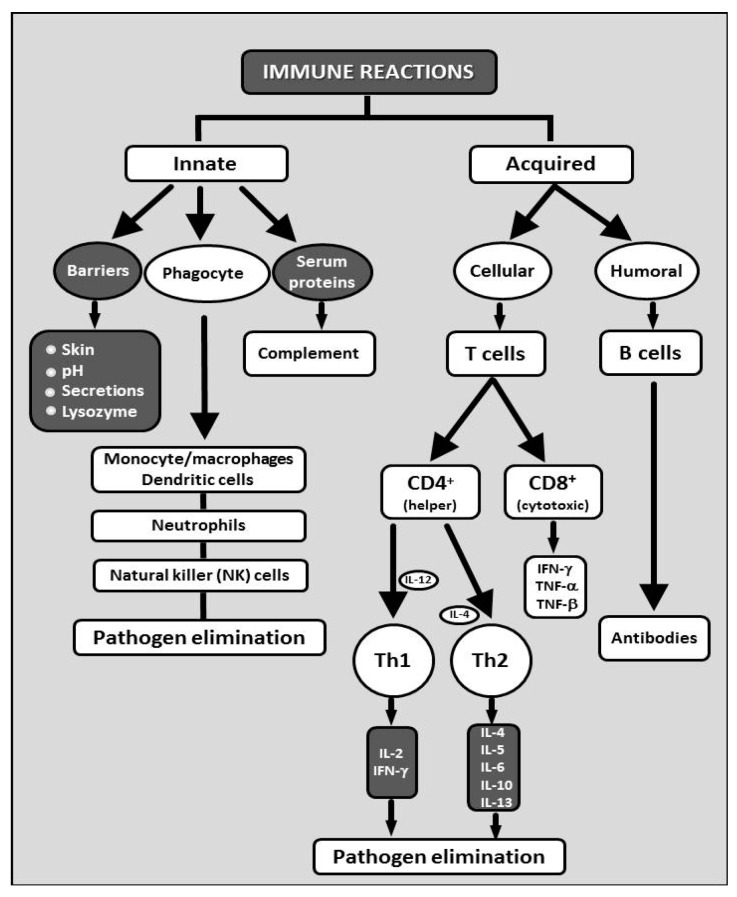
Various aspects of the both branches of immune reactions. Reaction known to be influenced by glucan are represented in white, reactions where glucan has no confirmed effects are shown in black.

**Figure 2:**
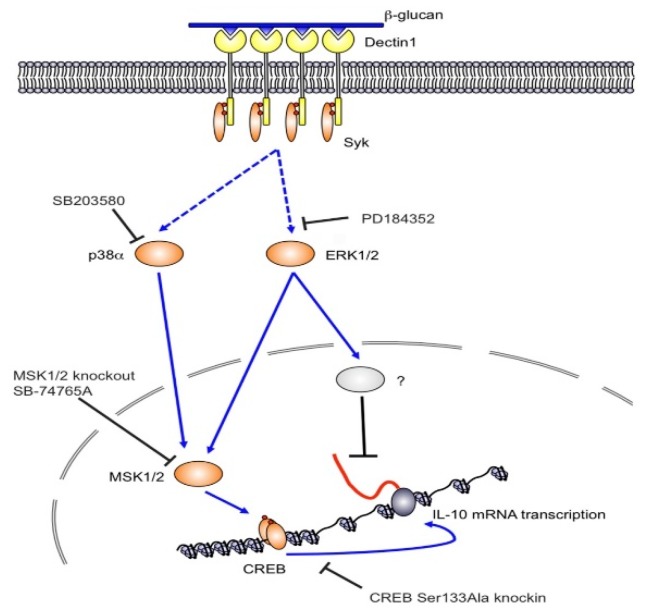
ERK1/2 regulate IL-10 transcription via MSK1/2 dependent and independent mechanisms. Dectin-1 is activated by complex β-glucan containing particles that induce clustering of dectin-1 at the membrane and formation of a phagocytic synapse. This leads to Syk recruitment to the ITAM like sequence in the cytoplasmic domain of dectin-1. Syk then mediates the activation of downstream signaling including the ERK1/2 and p38α MAPK cascades. Both ERK1/2 and p38α phosphorylate and activate the protein kinases MSK1 and 2. These in turn phosphorylate CREB on the IL-10 gene promoter, which stimulates IL-10 mRNA transcription. In addition, ERK1/2 also activates an MSK and p38 independent pathway that inhibits IL-10 mRNA transcription. In addition to the ERK1/2 and p38α pathways shown dectin-1 also activates NFκB that likely also plays a role in inducing IL-10 transcription. From (Elcombe et al., 2013) [50].

**Figure 3:**
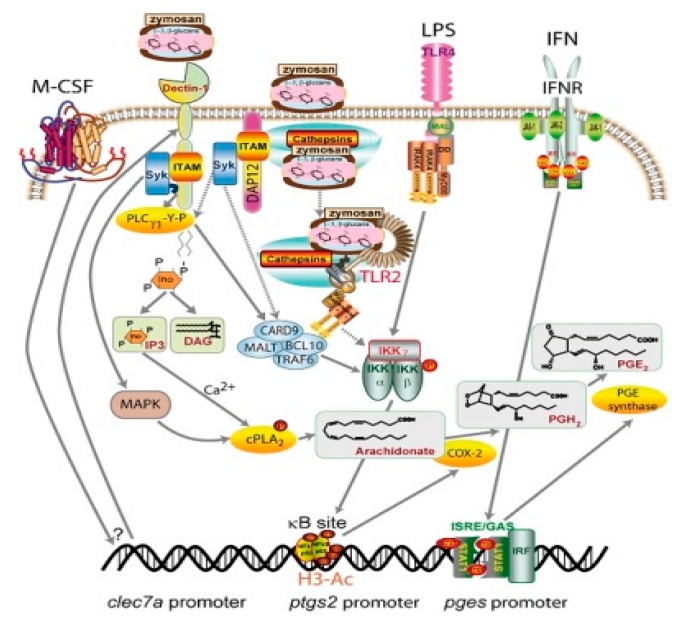
Proposed mechanisms involved in glucan/zymosan uptake and signaling in human macrophages. Zymosan particles can activate Syk via Dectin-1 engagement and via adaptor protein DAP12. Upon internalization, TLR2 recognition and cathepsin B leakage occur. In the presence of M-CSF, the expression of Dectin-1 B isoform is increased. Concomitant mechanisms of NF-κB activation might be triggered. cPLA_2_ is activated by Dectin-1/Syk-dependent mechanisms involving MAPK-dependent Ser-505 phosphotylation and Ca^2+^-dependent membrane translocation. Dotted lines indicate the steps associated with phagocytic cargo processing. Ac-H3 indicates acetylated histone 3. From (Municio et al., 2013) [51].

Glucans are known for significant effects on various branches of the immune system. The individual components of the immune system affected by glucan are summarized in Figure 1. Glucan is recognized by various receptors present on membranes of cells such as macrophages, monocytes, dendritic cells, and NK cells. The most important receptors are Dectin-1 and CR3 (CD11b/CD18); additional receptors include Toll-2, lactosylceramides, and the scavenger receptor family (see review, Vetvicka, Richter, Svozil, Rajnohova Dobiasova and Kral [22]). Upon binding, various processes occur, including direct receptor activation and/or cellular pathway activation [46]. A subsequent series of experiments demonstrated that glucans have strong pleiotropic effects on various parts of the defense apparatus including cytokine production and antibody responses [47,48]. Many reports described that glucan-activated B cells secrete some pro-inflammatory lymphokines (such as IL-8). This secretion required direct involvement of several molecules such as Dectin-1 receptors, mitogen-activated protein kinase (MAPK), and the transcription factors NF-κB and AP-1 [49]. Possible mechanisms for ERK1/2 regulation of IL-10 transcription are shown in Figure 2 [50]. Figure 3 suggests a slightly different mechanism of glucan involvement in signaling [51]. Clinical and physiological perspectives of glucan can be found in Bashir and Choi [52].

**Figure 4:**
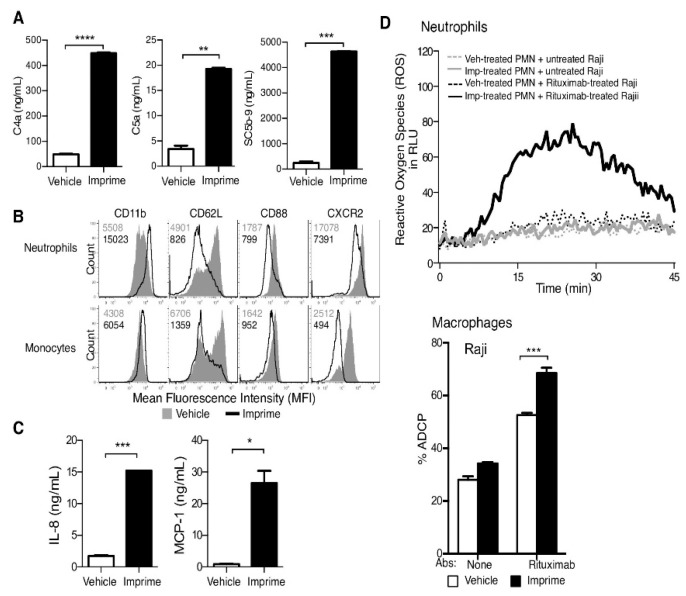
Imprime activates innate immune functions. (**A**) Complement activation proteins C4a, C5a, SC5b-9 in the plasma of whole blood treated with 10 μg/mL Imprime or vehicle for 30 min at 37 °C was measured by ELISA. Data represent mean ± SEM of triplicates for each treatment condition. (**B**) Modulation of CD11b, CD62L, CD88 and CXCR2 expression on neutrophils and monocytes post Imprime binding in whole blood was determined by flow cytometry. (**C**) Chemokine, IL-8 and MCP-1, production in the plasma of whole blood treated with Imprime or vehicle for 24 h at 37°C was measured by Luminex. Data represent mean ± SEM of duplicates from 3 independent experiments. (**D**) ROS production in 25:1 co-cultures of neutrophils (isolated from whole blood treated with 25 μg/mL Imprime or vehicle for 2 h at 37 °C) and Raji cells treated with or without 1 μg/mL rituximab was measured by luminescence-based assay. Macrophage-mediated ADCP was measured by flow cytometry after 1:1 co-incubation of macrophages (differentiated from monocytes isolated from WB treated with 25 μg/mL Imprime or vehicle for 2 h at 37 °C) with Raji cells treated with or without 1 μg/mL rituximab. Representative results are shown here from at least 3 independent experiments performed with three different donors (from Chan et al., 2016) [53].

As glucan can affect numerous facets of the immune response, it is not surprising that it also activates early innate reactions typically triggered by pathogen-associated molecules. Figure 4 summarizes the individual ways of glucan effects, from complement activation to neutrophil and monocyte activation [53]. For detailed information on the effects of glucan on immune reaction see [54]. Additional lesser-known effects of glucan include improvements in colitis, obesity, or Lyme disease [55,56,57].

## 3. Glucan in Dermatology

Effects of glucan in dermatology are less studied, probably due to the fact that commercial glucans are usually not soluble. In most cases, glucan is used in wound healing. When applied directly to the wounds, glucan significantly improved wound healing in diabetic mice, most probably via stimulation of macrophages [58]. Similar results were found in a rat model [59]. Mushroom-derived glucan was found to augment skin reaction induced by bradykinins, suggesting activation of endothelium via generation of vasoreactants and increase in sensitivity to vasoreactants [60]. In addition to wound healing, glucan is also used in cosmetic formulation, where it can act in oxidative stress and in improvement of various skin conditions, particularly skin moisture and skin microrelief [61]. A long-term use of glucan showed reduction of wrinkle depth, height and overall roughness [62], which is probably caused by stimulation of fibroblast and increase production of collagen.

## 4. Glucan in Clinical Trials

Since glucan therapy has achieved remarkable success in pre-clinical animal models, many efforts have been made to determine their therapeutic efficacy in human patients. In Japan, glucans derived from the Shiitake mushroom (lentinan) and from *Coriolus versicolor* (polysaccharide-K) have been licensed as successful drugs since 1983. Currently, the American database ClinicalTrials.gov summarizes 177 β-glucan clinical trials, mostly in cancer, gastrointestinal tract therapy, lowering cholesterol and improvements of immune reactions. In addition, this database mostly covers trials either performed in the USA or at least involving US companies, therefore the overall number of clinical trials currently running will be much higher. It is clear that it falls out of the scope of our review to go into any details. However, the most promising trials in cancer research involve the use of glucan together with monoclonal antibodies. Most of all, several clinical trials focusing on soluble glucan Imprime PGG in combination with the monoclonal antibodies are currently under way [63]. Clearly, glucan acts as a strong adjuvant for antibody therapy of cancer eliciting cell-mediated tumor-killing mechanisms that are not triggered by monoclonal antibody therapy alone. Even when glucan, used independently, demonstrated significant tumor regression, it was probably a result of cooperation with naturally occurring antitumor antibodies. The advantages of combined therapy with monoclonal antibodies or vaccines and glucan offer several advantages such as an easier elicitation of an antibody response, and a possible use of commercial humanized antibodies. Although data from most of these trials have not been released, a recent trial conducted by the Biothera using Imprime glucan plus Erbitux and chemodrug has released its clinical results and the data suggested that the combination of Imprime, Erbitux, and Camptosar nearly doubled the overall response rate of second- and third-line metastatic colorectal cancer patients compared with the drugs alone [64]. Among other combinations currently tested in clinical trials is glucan with Pemrolizumab and glucan with Atezolizumab and Bevacizumab (non-small cell lung cancer, colorectal cancer, and triple-negative breast cancer).

Another clinical trial studied the mushroom-derived glucan, Maitake, in myelodysplastic syndromes, which can progress to acute myelogenous leukemia [65]. The results showed elevated functions of neutrophils and monocytes, particularly production of reactive oxygen species. For more information about glucans and their application in cancer therapy, see Aleem [66].

**Figure 5:**
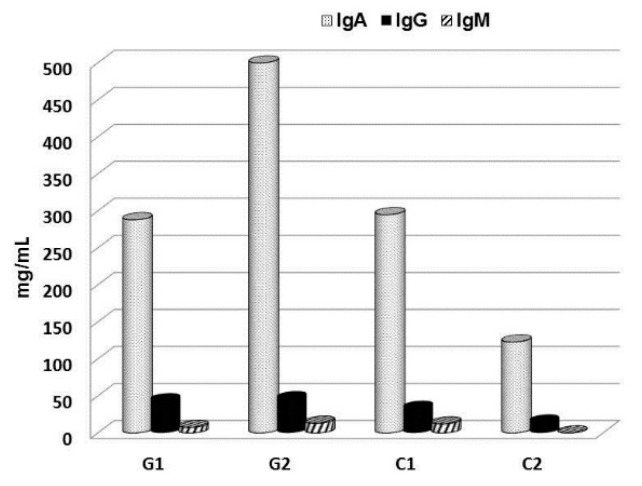
Changes in salivary immunoglobulins after 30 day supplementation in children with respiratory problems. G—glucan group, C—placebo group.

**Figure 6:**
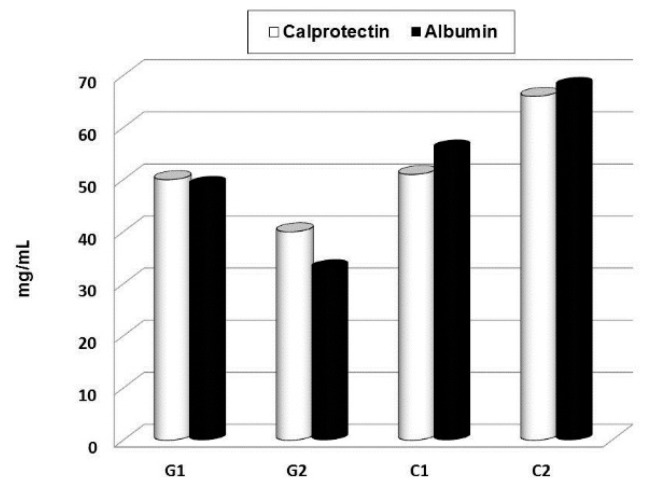
Effects if 30 days oral administration of glucan on calprotectin and albumin levels in saliva. G—glucan group, C—placebo group.

In parallel, our own research focused on children with chronic respiratory problems. In the last decade, we conducted a series of clinical, placebo-driven trials evaluating the effects of a short-term supplementation with glucan on the immune parameters in children. For these studies, we used Glucan #300, which is currently the most tested commercially available glucan with established high immunostimulating activity [67]. Randomly selected groups of children were treated with an oral dose with 100 mg of glucan/day for a period of 30 days. The results showed that this short-term supplementation improved the levels of salivary immunoglobulins (sIgM, sIgG, and sIgA) (Figure 5) [22], decreased eNO levels, and improved physical endurance of children [68]. Further, a short-term food supplementation with glucan also reduced the levels of salivary albumin and calprotectin (Figure 6). In addition, the same glucan treatment significantly improved the overall health status and physical conditions of children exposed to passive smoking [68,69]. These studies allowed us to conclude that glucan supplementation is a highly promising and inexpensive method of treatment for chronic respiratory problems in children [70]. Furthermore, a similar study on adults performed by an independent research group fully supported our findings [71].

**Figure 7:**
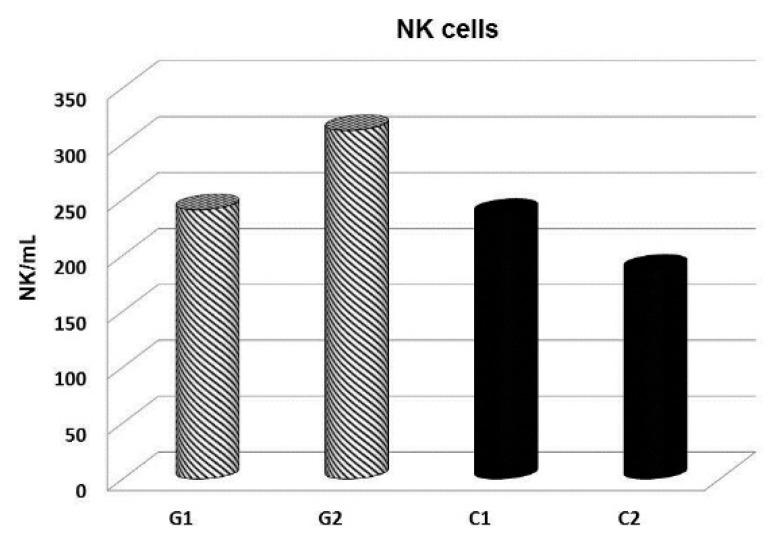
Total numbers of NK cells in peripheral blood of cancer patients before and after 60 day glucan supplementation. G—glucan group, C—placebo group.

In addition, some clinical trials performed focused on the effects of glucan supplementation on patients with cancer. Our studies showed that the addition of glucan to food stabilized IgG1 levels depressed by cancer therapy [72]. When the period of supplementation was increased to three months, significantly improved hematopoiesis was observed with the subsequent improvements of both physical and psychological conditions and reduction of possible complications resulting from subjective concerns about one’s own health. [73]. Further, a follow-up study found a significant increase of NK cells numbers and activity (Figure 7) suggesting that the addition of glucan to the diet could help the prevention of cancer remission. In addition, the glucan-supplemented group exhibited improvements in psychic conditions, cancer related fatigue, and nutritional state [68,74].

Efforts to regulate energetic metabolism, obesity, and/or metabolic syndrome are gaining the attention of scientists and medical professionals. The relationship between these conditions and glucan has been the focus of many human studies which have shown that glucan supplementation prevents or even treats metabolic syndrome and decreases insulin resistance, dyslipidemia, and obesity [75,76]. Clinical studies of diabetic retinopathy patients showed that dietary supplementation with vitamin D/glucan combination increased leptin levels [77,78]. Taken together with the current suggestions of leptin use in the treatment of diabetes, these reports are opening a new window in the treatment of this disease.

A systematic review of randomized controlled trials did not reach any conclusions on efficacy and safety of oral and inhalation commercial products [79], but for unexplained reasons this study reviewed only 30 studies.

## 5. Beta Glucan as Food Supplement

Food is the main source of substances necessary for growth and bodybuilding, as it contains the materials for renewal and regeneration of cells and tissues. It is also the only source of energy needed for physiological reactions, metabolism, and the immune response outside challenge. Apart from the basic components (such as proteins, lipids, sugars, vitamins, and minerals), the diet must contain non-digestible compounds, most of all dietary fibers.

Presently, dietary fiber is generally considered to be a prebiotic. Prebiotics were originally defined as non-digestible food ingredients that beneficially affect the host via stimulation of the growth or activity of bacteria present in the colon [80]. Foods high in prebiotics, including glucans forming structural components of cell walls, have been commonly consumed since prehistoric times. This plant-based diet helped to influence human genes involved in digestive metabolism and food utilization long before men diet has shifted to meat-based diets. These genes were specific for the direct predecessors of both hominids and hominins. Hominins are part of the family, or larger group of primates, called hominids, which includes orangutans, gorillas, chimpanzees, and human beings. All hominins are hominids, but very few hominids are hominins, and are involved in the nutritional behavior of modern humans. For more information about the dietary fibers and their transport across the gut, see de Jesus Raposo, et al. [81].

Numerous studies describing not only the beneficial health effects of glucan, but also nutritional benefits of glucan, represent the basis for possible use of these substances as novel pharmaceuticals [82]. Further, a study using fishes showed that glucan supplementation, tested as potential probiotic, supported the activity of *Lactobacillus spp.* and strongly decreased mortality of tilapia from *Aeromonas spp.* challenge [83]. However, more attention has been paid to prebiotics than to probiotics. A surprising study revealed that simultaneous application of glucan and starch during cold storage significantly increased survival of bifidobacteria strains in yogurt, most probably due to the protective effects on bifidobacteria stress caused by low temperatures [84]. Study results suggest that beta-glucan has a protective effect on bifidobacteria in yogurt, the effects of starch were minimal. Similar effects were obtained in shrimp and calves—use of glucan as a prebiotic positively influenced some humoral immunological parameters such as the total protein and immunoglobulin concentration [85].

Furthermore, glucan not only affects the immune system but may also reduce cholesterol levels. In fact, major physicochemical properties of glucan include their antioxidant effects, which are involved in the scavenging of reactive oxygen species and their role as dietary fiber subsequently preventing the absorption of cholesterol [86]. Previously, it was considered that soluble dietary fiber might lower levels of lipids and cholesterol in the blood and that insoluble dietary fiber only contributed to fecal bulking. However, the hypocholesterolemic effects of dietary fiber are still not fully understood. Therefore, the effects were attributed to the ability of soluble dietary fiber to form viscous solutions that prolong gastric emptying, inhibit the transport of triglycerides and cholesterol across the intestine, and reduce total low density lipoprotein concentrations [87]. Additionally, the hypocholesterolemic effects of glucans were confirmed in broiler chicks, which were fed a diet supplemented with glucan-rich barley [88]. Lim et al. [89] applied glucans from yeast-like fungus *Aureobasidium pullulans* to a hamster experimental animal model of hyperlipemia induced by a high-fat diet. In a rat experimental model, glucan reduced total cholesterol by 32% triglyceride by 64%, and malondialdehyde levels by 45% [90]. Moreover, in a study carried out by the current authors, yeast-derived glucan #300 markedly lowered total cholesterol levels in mice with experimentally-induced hypercholesterolemia [69]. In addition, supplementation with three other glucans, Krestin, ImmunoFiber, and Now glucan, induced similar, albeit less significant effects [48]. Further, long-term clinical studies investigating the effects of soluble forms of glucans in men with hypercholesterolemia demonstrated that they decrease blood cholesterol levels [91,92]. In fact, studies published over the past 20 years have demonstrated that glucan effectively reduced low density lipoprotein cholesterol levels following the supplementation of diets with glucan in humans with high blood concentrations of triglycerides and cholesterol [93,94]. Nevertheless, further research is required to specifically define the role of glucan in modulating the immunological aspects of atherosclerosis, as the exact mechanisms of these glucan actions are still not established. With the superior effects of glucan, the recent reviews strengthened the hypotheses that of all the forms of dietary fiber, natural glucan molecules are the most promising to use as a method of treating patients with dyslipidemia [55]. However, it remains unclear whether increasing glucan intake should be recommended for patients with severe hypercholesterolemia, to be used in addition to or even as an alternative to statins, as there have been very few direct comparisons (see review by Sima, et al. [95]).

## 6. Transport through the Gut

Detailed information about the mechanisms of transport of glucan through the gastrointestinal tract is still far from clear. For some time, it was even suggested that orally-administered glucan could not have any activity, because the human gastrointestinal tract lacks the enzyme necessary to break down the glucan molecule. This false hypothesis was pushed around without any scientific proof and was later corrected by studies directly comparing the effects of injected and orally used glucan [96]. Later studies revealed that the main route for a particular antigen to the body is through M cells localized within Peyer patches, which are considered to be the main site of entry, largely due to their intimate localization with the intestinal lumen. In fact, the original suggestion that insoluble glucans are taken up by Peyer patches M cells goes back almost 30 years [97].

Availability of soluble glucan allowed more direct studies on gastrointestinal transport. A detailed study showed that the speed of transfer through the gut differs based on the type of glucans used, ranging from 0.5–12 h. The speed of transfer depends on the physicochemical characteristics of glucan. A flow analysis of the glucan presence showed that after oral administration, glucan could be found inside the cells after 24 h. The first cells able to engulf glucan were intestinal epithelial cells. As only 10% of them can internalize glucan, it is possible that only a special subpopulation of intestinal cells participate in glucan transfer [98]. We would like to stress that glucan does not represent essential nutrients, but it might be successfully used not only for improvement of immune functions but also to improve the general quality of life via improvements of immune status, lowering cholesterol, improving blood glucose levels and reduction of stress.

## 7. Glucan and Medicine

Glucans are always isolated from natural sources. They represent molecules that can bind to specific receptors on individual subsets of the immune cells. As the stimulation of various facets of the immune reaction is well-established, it is not surprising that various glucans are currently being tested in several types of diseases known for the immune system dependence.

In addition to the direct treatment, there is also substantial research on the use of glucan in drug delivery carrier systems, both as part of a new drug or in vaccines, or even as a targeting molecule. One of the currently-tested possibilities is glucan-based nanoparticle systems. In those systems, the insoluble glucans can be prepared as hollow spheres, which may carry various types of compounds, with the outer shell capable of binding to cells via specific receptors. Hence, as therapeutically important payload of biological agents such as siRNA, peptides and DNA can be encapsulated, these delivery systems represent a novel and highly promising tool. Glucans can be used as novel hydrogels or as direct immunocyte-targeting delivery systems employing novel complexes with oligodeoxynucleotides or offer a new approach to immunization and cancer immunotherapy (see review, Zhang, et al. [99]).

Additionally, most of the recent research has focused on yeast-derived glucans. Glucan derived from *Saccharomyces cerevisiae* can be processed into hollow and highly porous microparticles. Their use goes back more than 25 years ago when Alpha-Beta developed glucan carbohydrate microcapsules (Adjuvax) for specific antigen and drug delivery. The use of Adjuvax resulted in more than a 1000-fold increase in antibody response [100]. Recently, glucan particles have been successfully used to prepare encapsulated polyplexes for DNA, siRNA, and proteins [101,102]. Another study showed the highly effective use of glucan-encapsulated siRNA particles as an orally-delivered material to silence genes in mouse macrophages in vivo [103]. This possibility was further confirmed by preparation of glucan microparticles for mucosal antigen delivery. The oral application of these microparticles increased antigen-specific IgA, secretory-IgA and secretory component production in intestinal fluids and induced specific antibody formation via efficient antigen presentation and Th17-biased response [104]. In addition, it triggered the expression of pro-inflammatory cytokines IL-23p19, IL-8 and the β-glucan receptors Dectin-1 and TLR2. Similar results were found in additional experimental models, such as the case of experimental vaccination against cryptococcosis, where the particles protected mice against cryptococcosis in a manner dependent upon mouse strain and cryptococcal species [105] or HBsAg where it increased the antibody formation [106]. In another study, glucan was successfully used as an adjuvant, with excellent results in a mouse model of H5N1 vaccine [107].

Intensive research done by Ross and his colleagues used glucan simultaneously with antitumor antibodies [43]. This approach offers several important advantages: external antibodies supplement the low antibody production during cancer and act as targeting molecules, resulting in great synergy and improved effects. It is important to stress that any antitumor antibodies able to activate complementarily can be combined with glucan, as glucan has no preference between antibodies [42].

The role of glucan in allergies is still not clear. On one hand, glucan is a common structural component in a variety of allergens. On the other hand, several recent studies suggested that glucan plays a role in alleviation of allergic problems [41,108]. Despite the documented effects of glucan in allergies, the possible mechanisms are not yet fully established. Most hypotheses assume that these effects are manifested via decreasing proinflammatory cytokines (mostly IL-6 and TNF-α) and increased formation of antioxidants [109,110].

Additionally, based on in vitro and in vivo studies documenting the suppressive effects of glucan on experimentally induced allergic reactions in animal models [41,111], several clinical trials applied glucans to patients suffering from asthma or other allergies [112]. One of the first studies on the therapeutic use of glucan for asthma involved an *Agaricus blazei-*derived glucan in treatment of a bronchitic patient with asthma-like symptoms [113]. After oral supplementation for 60 days, the attenuation of bronchitic symptoms was observed accompanied by changes in Th2-dependent IL-10 and Th1-dependent IFN-γ production. In another study, four weeks of glucan supplementation resulted in alleviated symptoms and increased physical health. Surprisingly, the serum levels of IgE remained unchanged [114]. Some authors affirmed that glucan supplementation can improve, or even prevent, symptoms of allergic rhinitis and upper respiratory tract infections [115,116], as well as some other allergic symptoms such as elevated production of IgE antibodies [117,118].

In the study by Sarinho et al., dealing with asthmatic children, particulate glucan was applied subcutaneously [119], resulting in the increased production of anti-inflammatory IL-10 and alleviated asthmatic symptoms. In addition, research showing that the glucan-resveratrol combination has beneficial effects in children with allergic rhinitis clearly supports these data [120,121], since that leads to shorter episodes of respiratory infections and less need of antibiotics [122]. For more details on glucan and allergies, see recent review by Sima and Vetvicka [108].

Moderate physical activity is clearly beneficial not only to our health, but also to our immune system. However, excessive physical challenges are known to decrease the quality of immune reactions. In elite athletes, food supplementation with glucan improved exercise-related drop in NK cell functions and cell counts [123]. In addition, glucan is often used as a nutritional supplement, protecting athletes against upper respiratory infections [124]. Using mice models, Murphy’s group demonstrated that beneficial effects of glucan are manifested via lung macrophages [125]. This hypothesis was evidenced by the fact that depletion of lung macrophages negated the beneficial effects of glucan. However, human studies suggested that glucan supplementation offsets the increased risk of urinary tract infections associated with exercise stress, probably via stimulation of lung macrophages [126].

## 8. Conclusions

Glucans undoubtedly occupy a prominent position among immunomodulators. They are clearly defined by their origin and structure. In addition, they are relatively inexpensive and possess extremely low risk of negative side effects. The effects of glucan on a variety of diseases, including infections, arthritis, diabetes, low immunity and cancer, have been investigated. However, glucans are still criticized, often by nonprofessionals, because of insufficiently defined preparations and unclear or nonspecific effects. Fortunately, in the last 15 years, research in reputable laboratories from all around the world has finally reached the stage where the basic mechanisms of glucan effects are well established and individual activities have been clearly explained. It is our belief that glucan will soon hold the position ascribed more than four decades ago.
